# A Study of the Quality of Life in Patients on Hemodialysis Therapy

**DOI:** 10.7759/cureus.94864

**Published:** 2025-10-18

**Authors:** Diljeet Bodra, Punam Munda, Bhargavi Bhojaraju, Mashud Mohd Essar Hussain Khan

**Affiliations:** 1 Nephrology, Rajiv Gandhi University of Health Sciences, Bengaluru, IND; 2 Medicine, Netaji Subhash Chandra Bose Hospital, Jamshedpur, IND; 3 Nephrology, Father Muller Medical College, Mangalore, IND

**Keywords:** chronic kidney disease (ckd), hemodialysis (hd), nutritional status, psychological well-being (pwb), quality of life, whoqol-bref

## Abstract

Aim: To study and compare demographic data, medical parameters, and quality of life (QoL) using the World Health Organization Quality of Life-BREF (WHOQOL-BREF) tool among hemodialysis (HD) patients at Father Muller Medical College Hospital (FMMCH), Mangalore.

Methods: This cross-sectional study was conducted over a period of six months among 100 adult patients with end-stage kidney disease (ESKD) undergoing maintenance HD at FMMCH. Data were collected using a structured questionnaire comprising demographic information, clinical parameters, and the WHOQOL-BREF instrument to assess four domains of QoL: physical, psychological, social, and environmental. Statistical analyses were carried out using appropriate tests, including Student’s T-test and one-way ANOVA, with a p-value of <0.05 considered statistically significant.

Results: Among 100 HD patients, the mean physical domain score was 54.4 ± 9.7, and the psychological domain score was 56.6 ± 10.8, indicating that these areas were most impaired. In contrast, social and environmental domains were relatively preserved (55.1 ± 9.1 and 60.8 ± 11.2, respectively). QoL in the social domain improved significantly with longer dialysis duration (p = 0.01), and physical domain scores varied significantly with income (p = 0.02). Multiple regression confirmed dialysis duration as a significant independent predictor of overall QoL (β = 2.81, p = 0.017).

Conclusions: The research found that maintenance HD patients had experienced a suboptimal QoL with considerable physical and psychological deficits. Income was highly linked to physical health, whereas dialysis duration was favorably related to social well-being.

## Introduction

Chronic kidney disease (CKD) is a worldwide health issue characterized by the gradual deterioration of kidney function, often culminating in end-stage kidney disease (ESKD). At this juncture, renal replacement treatment, via either hemodialysis (HD), peritoneal dialysis (PD), or kidney transplantation, is critical for the preservation of life [1]. In India, where not everyone can get a transplant and organ donation rates are still low, HD is still the most common way to treat ESKD [2]. HD is a therapy that keeps people alive and is normally administered three times a week for four hours each time. But it also imposes a significant physical and psychological burden [3].

Globally, the incidence of ESKD is estimated to range between 350 and 400 cases per million population annually, with over two million people receiving dialysis worldwide [4,5]. In India, by comparison, there are an estimated 229 new cases of ESKD per million population each year, and more than 100,000 patients initiate dialysis annually [4]. These figures highlight both the substantial burden of kidney failure in India and the gap in access to renal replacement therapy compared with high-income countries. Beyond survival, patients face significant physical and psychological challenges that hinder daily functioning, making the maintenance of quality of life (QoL) as critical a goal as prolonging life itself [6].

QoL, according to the World Health Organization (WHO), is how an individual views their living circumstances in light of their goals, ambitions, and cultural and value systems [7]. Several factors affect QoL during HD, such as comorbidities, nutritional status, mental health, socioeconomic situations, and access to healthcare services. Malnutrition and protein-energy wasting are common in HD patients, resulting in increased morbidity and mortality rates [8]. Additionally, psychological stress, the forfeiture of social obligations, and reduced autonomy stemming from extended treatment durations and chronic illness may further aggravate the deterioration of QoL [9].

Educational interventions, particularly those focused on nutrition, illness management, and self-care, have shown effectiveness in improving patient outcomes and adherence [10]. Adequate guidance on fluid intake and the regulation of phosphorus, sodium, and potassium can help prevent complications such as renal osteodystrophy, cardiovascular events, and hospital readmissions. However, patient education remains suboptimal, particularly in resource-limited settings [11].

To identify problem areas, the research must assess QoL in a systematic and trustworthy manner. To evaluate the four components of QoL: mental health, physical health, social relationships, and environmental variables, the WHO developed the World Health Organization Quality of Life-BREF (WHOQOL-BREF) questionnaire [12-14]. Researchers often utilize this instrument to learn more about how patients with chronic diseases feel and how their medications impact them [15].

An increasing number of patients in regions such as Mangalore, India, require dialysis, particularly at tertiary centers like Father Muller Medical College Hospital (FMMCH). Still, there is little evidence that HD affects their QoL. Looking into this part of the issue might assist in filling in information gaps and stimulate the development of comprehensive, patient-centered treatment programs. This research seeks to evaluate and analyze the QoL in patients receiving maintenance HD at FMMCH, Mangalore, with the WHOQOL-BREF questionnaire, hence improving the comprehension and progression of treatment methods for this susceptible group.

## Materials and methods

Study design

A cross-sectional study was conducted in the Nephrology Department of FMMCH, a tertiary care teaching facility located in Mangalore, Karnataka, India. The protocol was submitted for ethical review in March 2024, and approval was granted on 15 July 2025 by the Institutional Scientific and Ethical Committee. Preparatory activities, including tool translation and pilot testing, were undertaken in advance. Patient recruitment and data collection were carried out within a two-month period between July and August 2025, immediately following approval.

Study population

The study included 100 adult patients (≥18 years) with a confirmed diagnosis of ESRD, who had been receiving HD for a minimum of three months and could provide informed written permission. Only patients who were compliant with their prescribed HD schedule were included, as verified from dialysis unit records. Information regarding the clinical history and primary causes associated with ESRD, including diabetes mellitus (DM), hypertension (HTN), ischemic heart disease (IHD), and polycystic kidney disease (PKD), as well as dialysis-related parameters such as frequency (twice or thrice weekly) and duration of dialysis (dialysis vintage), was documented for all participants. People who had current cancers, had significant surgery in the last six months, or had cognitive problems that would make it hard for them to answer questions were not allowed to participate. The sample size of 100 was calculated using G*Power software (version 3.1.9.7, Heinrich-Heine-Universität Düsseldorf, Düsseldorf, Germany), assuming a medium effect size (Cohen’s d = 0.5), 80% statistical power, and a significance level of 5% (α = 0.05).

Questionnaire tool (WHOQOL-BREF)

The WHOQOL-BREF questionnaire, a condensed version of the WHOQOL-100 that has been authorized by the WHO, was used to gauge the participants' level of life satisfaction. Physical Health (items 3, 4, 10, 15, 16, 17, 18), Psychological Health (items 5, 6, 7, 11, 19, 26), Social Relationships (items 20, 21, 22), and Environmental Health (items 8, 9, 12, 13, 14, 23, 24, 25) are the four primary areas into which this instrument is divided. Each problem was evaluated using a five-point Likert scale, and the raw data were transformed into a standardized 0-100 scale, with higher values signifying an enhanced QoL. Because WHOQOL-BREF is adaptable and applicable to a wide range of demographics and situations, it is especially suitable for assessing the QoL of patients with end-stage renal disease receiving continuous HD [16].

Data collection tools and procedures

Data collection was accomplished using a pre-structured questionnaire available in both English and Kannada, facilitated by semi-structured, face-to-face interviews carried out by trained interviewers who were independent of the dialysis unit personnel to mitigate any bias. The questionnaire consisted of three primary components: a quality-of-life evaluation, medical information, and demographic data. The demographic information included age, gender, marital status, education, occupation, income level, and socioeconomic position (as measured by Kuppuswamy's scale) [17]. Higher education, monthly earnings for families, and occupation were all included in the revised Modified Kuppuswamy's Socioeconomic Status Scale (2024 edition), with income categories modified in accordance with the current year's Consumer Price Index (CPI). Clinical history, including length of diabetes or HTN, frequency and duration of HD, and family history of ESKD; relevant laboratory results, including hemoglobin, serum urea, and bloodstream creatinine; and the presence of several medical conditions, such as PKD, coronary artery disease, HTN, and DM, were among the medical parameters covered. Literate patients completed the questionnaire independently, while illiterate participants were assisted by the interviewer in recording their responses without influencing them. Given the use of interviewer-assisted administration for illiterate participants, there is a possibility of response bias, especially for sensitive items such as sexual satisfaction and emotional well-being. To minimize this, interviewers were trained to maintain neutrality, ensure confidentiality, and conduct interviews in private settings whenever possible. Nonetheless, the influence of social desirability bias cannot be entirely excluded.

Ethical considerations

The study protocol was reviewed and approved by the FMMCH, Mangalore's Institutional Ethics Committee (FMIEC/CCM/637/2025). All patients were informed of the study's goals and methods prior to their participation, and each participant provided signed informed consent.

Statistical analysis

IBM SPSS Statistics for Windows, Version 26 (Released 2018; IBM Corp., Armonk, New York, United States) was used to assemble and analyze the study's data. QoL ratings, clinical factors, and demographics were summarized using descriptive statistics, including means, standard deviations, frequencies, and percentages. One-way analysis of variance (ANOVA) was used for analyses involving more than two groups, and Student's t-test was used for comparisons comprising two groups to investigate the association between categorical factors and WHOQOL-BREF domain scores. To address the risk of Type I error due to multiple subgroup comparisons, a Bonferroni correction was applied where appropriate. P-values below 0.05 were regarded as statistically significant. Data was checked for missing or insufficient replies before analysis. Participants with partially completed WHOQOL-BREF domains were excluded from domain-specific analysis, while cases with minor missing values (<5%) were managed using pairwise deletion to preserve statistical power and reduce bias.

## Results

Demographic characteristics

The demographic and clinical characteristics of the HD patients (N = 100) reveal that the majority were aged 60 years or older (52%) and mostly male (69%). Many participants lived in semi-urban settings (65%) and were married (85%). The level of education varied: 38% had just basic school, and 35% had graduated. Around 53% of people worked in the private sector, and 32.9% said they made between ₹1 and ₹5 lakhs a year. Clinically, 75% had been on dialysis for more than one year, 85% had diabetes for more than ten years, and 87% had high blood pressure for more than five years. Around 11% of the people had IHD, and two percent had PKD. Most people needed dialysis two (60%) or three (41%) times a week, which shows that they were quite dependent on the therapy (Table 1).

**Table 1:** Demographic characteristics of hemodialysis patients

Demographic characteristics	Categories	Frequency (N)	Percentage (%)
Age (in years)	18 - 34	8	8.0
	35 - 60	40	40.0
	≥ 60	52	52.0
Gender	Male	69	69.0
	Female	31	31.0
	Total	100	100.0
Residence	Urban	24	24.0
	Semi Urban	65	65.0
	Rural	11	11.0
Marital Status	Married	85	85.0
	Unmarried	15	15.0
Education	Illiterate	14	14.0
	Primary	38	38.0
	Secondary/HS	13	13.0
	Graduation	35	35.0
Occupation	Government Job	5	5.0
	Private Job	53	53.0
	Daily Wage	18	18.0
	Housewife	24	24.0
Income (Rs. In Lakhs)	≤ 1	13	17.1
	1 - 5	25	32.9
	5 - 10	22	28.9
	≥ 10	16	21.1
Duration of Dialysis	≤ 1	25	25.0
	1 - 5	40	40.0
	≥ 5	35	35.0
Diabetes Mellitus	< 10	15	15
	≥ 10	85	85
Hypertension	≤ 5	13	13
	> 5	87	87
Ischemic Heart Disease	Yes	11	11.0
	No	89	89.0
Polycystic Kidney Disease	Yes	2	2.0
	No	98	98.0
Twice Dialysis	Yes	59	60.0
	No	41	40.0
Thrice Dialysis	Yes	41	41.0
	No	59	59.0

Clinical characteristics

The clinical profile of the 100 patients (Table 2) shows that the average length of time they had been on dialysis was 5.61 months, which means they were in the early to middle stages of therapy. The average HD score was 1.36, which means that the patient was getting regular dialysis therapy. DM (mean: 13.15) and HTN (mean: 8.94) were the most frequent comorbidities, whereas IHD (mean: 0.49) was less common. These results highlight the significant prevalence of metabolic problems in dialysis patients.

**Table 2:** Descriptive statistics of clinical characteristics among study participants SD: standard deviation

Clinical characteristics	Mean	SD
Duration of Dialysis	5.6089	3.52588
Hemodialysis	1.3564	.54008
Diabetes Mellitus	13.1485	4.80080
Hypertension	8.9406	4.13236
Ischemic Heart Disease	0.4851	1.61006

Quality of life scores by WHOQOL-BREF domains

The responses to the WHOQOL-BREF questionnaire indicate that many participants rated their overall QoL positively, with 67% reporting it as good and 69% expressing satisfaction with their health. However, 88% reported experiencing physical pain to a moderate or greater extent, and 91% indicated a moderate to extreme need for medical treatment, reflecting significant health burdens. Emotional well-being was moderately preserved, with 54% enjoying life moderately and 58% finding life meaningful, though 71% experienced negative feelings quite often, suggesting prevalent psychological distress. Social and environmental factors were generally favorable, with high satisfaction in areas such as personal relationships (75% neutral), living conditions (52% satisfied), and access to health services (51% satisfied). Energy levels and ability to perform daily activities were moderate, while only 2% reported being very satisfied with their sex life, indicating a potential area of concern. Overall, while QoL was rated positively in several domains, pain, medical dependence, and emotional health emerged as key challenges (Table 3).

**Table 3:** Participants' responses to the WHOQOL-BREF questionnaire WHOQOL: World Health Organization Quality of Life

Questions	Responses	Frequency (N = 100)	Percentage
How would you rate your quality of life?	Very poor	-	-
	Poor	-	-
	Neither poor nor good	33	33.0%
	Good	67	67.0%
	Very good	-	-
How would you rate your quality of life?	Very dissatisfied	-	-
	Dissatisfied	5	5.0%
	Neither satisfied nor dissatisfied	25	25.0%
	Satisfied	69	69.0%
	Very satisfied	1	1.0%
To what extent do you feel that physical pain prevents you from doing what you need to do?	Not at all	3	3.0%
	A little	8	8.0%
	A moderate amount	38	38.0%
	Very much	50	50.0%
	An extreme amount	1	1.0%
How much medical treatment do you need to function in your daily life?	Not at all	-	-
	A little	9	9.0%
	A moderate amount	38	38.0%
	Very much	49	49.0%
	An extreme amount	4	4.0%
How much do you enjoy life?	Not at all	2	2.0%
	A little	9	9.0%
	A moderate amount	54	54.0%
	Very much	34	34.0%
	An extreme amount	1	1.0%
To what extent do you feel your life is meaningful?	Not at all	1	1.0%
	A little	10	10.0%
	A moderate amount	58	58.0%
	Very much	31	31.0%
	An extreme amount	-	-
How well are you able to concentrate?	Not at all	-	-
	A little	12	12.0%
	A moderate amount	39	39.0%
	Very much	4	4.0%
	Extremely	2	2.0%
How safe do you feel in your daily life?	Not at all	1	1.0%
	A little	6	6.0%
	A moderate amount	46	46.0%
	Very much	47	47.0%
	Extremely	-	-
How healthy is your physical environment?	Not at all	-	-
	A little	9	9.0%
	A moderate amount	44	44.0%
	Very much	47	47.0%
	Extremely		
Do you have enough energy for everyday life?	Not at all	2	2.0%
	A little	10	10.0%
	A moderate amount	46	46.0%
	Very much	42	42.0%
	Extremely	-	-
Are you able to accept your bodily appearance?	Not at all	1	1.0%
	A little	11	11.0%
	A moderate amount	49	49.0%
	Very much	39	39.0%
	Extremely	-	-
Do you have enough money to meet your needs?	Not at all	2	2.0%
	A little	6	6.0%
	A moderate amount	43	43.0%
	Very much	48	48.0%
	Extremely	1	1.0%
How available is the information that you need in your day-to-day life?	Not at all	-	-
	A little	9	9.0%
	A moderate amount	45	45.0%
	Very much	45	45.0%
	Extremely	1	1.0%
To what extent do you have the opportunity for leisure activities?	Not at all	-	-
	A little	11	11.0%
	A moderate amount	48	48.0%
	Very much	41	41.0%
	Extremely	-	-
How well are you able to get around?	Very poor	-	-
	Poor	8	8.0%
	Neither poor nor good	41	41.0%
	Good	51	51.0%
	Very good	-	-
How satisfied are you with your sleep?	Very dissatisfied	-	-
	Dissatisfied	6	6.0%
	Neither satisfied nor dissatisfied	32	32.0%
	Satisfied	59	59.0%
	Very satisfied	3	3.0%
How satisfied are you with your ability to perform your daily living activities?	Very dissatisfied	-	-
	Dissatisfied	8	8.0%
	Neither satisfied nor dissatisfied	40	40.0%
	Satisfied	51	51.0%
	Very satisfied	1	1.0%
How satisfied are you with your capacity for work?	Very dissatisfied	-	-
	Dissatisfied	9	9.0%
	Neither satisfied nor dissatisfied	43	43.0%
	Satisfied	46	46.0%
	Very satisfied	2	2.0%
How are you with yourself?	Very dissatisfied	-	-
	Dissatisfied	2	2.0%
	Neither satisfied nor dissatisfied	60	60.0%
	Satisfied	38	38.0%
	Very satisfied	-	-
How satisfied are you with your personal relationship?	Very dissatisfied	-	-
	Dissatisfied	4	4.0%
	Neither satisfied nor dissatisfied	75	75.0%
	Satisfied	17	17.0%
	Very satisfied	4	4.0%
How satisfied are you with your sex life?	Very dissatisfied		
	Dissatisfied	13	13.0%
	Neither satisfied nor dissatisfied	85	85.0%
	Satisfied	2	2.0%
	Very satisfied	-	-
How satisfied are you with the support you get from your friends?	Very dissatisfied	2	2.0%
	Dissatisfied	2	2.0%
	Neither satisfied nor dissatisfied	43	43.0%
	Satisfied	51	51.0%
	Very satisfied	2	2.0%
How satisfied are you with the condition of your living place?	Very dissatisfied	-	-
	Dissatisfied	2	2.0%
	Neither satisfied nor dissatisfied	43	43.0%
	Satisfied	52	52.0%
	Very satisfied	3	3.0%
How satisfied are you with your access to health services?	Very dissatisfied	-	-
	Dissatisfied	3	3.0%
	Neither satisfied nor dissatisfied	46	46.0%
	Satisfied	51	51.0%
	Very satisfied	-	-
How satisfied are you with your transport?	Very dissatisfied	-	-
	Dissatisfied	2	2.0%
	Neither satisfied nor dissatisfied	49	49.0%
	Satisfied	47	47.0%
	Very satisfied	2	2.0%
How often do you have negative feelings, such as a blue mood, despair, anxiety, or depression?	Never	5	5.0%
	Seldom	15	15.0%
	Quite Often	71	71.0%
	Very often	8	8.0%
	Always	1	1.0%

Association between demographic factors and QoL domains

Comparison of WHOQOL-BREF Domain Scores Across Different Age Groups

There were no statistically significant variations in any of the WHOQOL-BREF domain ratings when comparing them across age groups (p > 0.05). However, participants aged 18-34 years had the highest mean physical (57.14) and psychological (57.81) scores, while those ≥60 years scored highest in the environment domain (61.78) and social relationships domain (55.45) (Table 4).

**Table 4:** Mean comparison of the WHOQOL-BREF domain with age SD: standard deviation, N: number of patients; WHOQOL: World Health Organization Quality of Life

Domains	Age	N	Mean	SD	One-way ANOVA
Physical Domain	18 - 34	8	57.14	12.37	0.55
	35 - 60	40	53.39	8.44	
	≥ 60	52	54.95	10.35	
Psychological Domain	18 - 34	8	57.81	7.20	0.90
	35 - 60	40	56.15	11.97	
	≥ 60	52	56.89	10.43	
Social Relationships Domain	18 - 34	8	52.08	5.89	0.62
	35 - 60	40	55.21	9.38	
	≥ 60	52	55.45	9.32	
Environment Domain	18 - 34	8	60.55	9.73	0.59
	35 - 60	40	59.38	12.68	
	≥ 60	52	61.78	10.09	

Comparison of WHOQOL-BREF Domain Scores Based on Gender

The WHOQOL-BREF domain ratings did not significantly vary by gender, according to the study (p > 0.05). Males had somewhat higher mean scores in the social interactions and environment domains, whereas females had slightly greater mean scores in the psychological (58.20 vs. 55.98) and physical (55.76 vs. 53.93) domains (Table 5).

**Table 5:** Mean comparison of the WHOQOL-BREF domain with gender SD: standard deviation, N: number of patients; WHOQOL: World Health Organization Quality of Life

Domains	Gender	N	Mean	SD	One-way ANOVA
Physical Domain	Male	69	53.93	10.21	0.39
	Female	31	55.76	8.68	
Psychological Domain	Male	69	55.98	10.80	0.34
	Female	31	58.20	10.78	
Social Relationships Domain	Male	69	55.31	7.69	0.71
	Female	31	54.57	11.75	
Environment Domain	Male	69	60.69	10.30	0.97
	Female	31	60.79	12.96	

Comparison of WHOQOL-BREF Domain Scores According to Educational Level

The results showed that there was no statistically significant correlation (p > 0.05) between WHOQOL-BREF domain scores and educational status. However, compared to other groups, participants with secondary or high school education had the greatest mean scores in the psychological (61.22) and physical (58.52) categories, whereas those who were illiterate indicated higher scores in the environment (63.39) and social interactions (58.33) domains (Table 6).

**Table 6:** Mean comparison of the WHOQOL-BREF domain with education SD: standard deviation, N: number of patients; WHOQOL: World Health Organization Quality of Life

Domains	Education	N	Mean	SD	One-way ANOVA
Physical Domain	Illiterate	14	55.36	13.05	0.409
	Primary	38	53.76	8.59	
	Secondary/HS	13	58.52	10.26	
	Graduation	35	53.47	9.31	
Psychological Domain	Illiterate	14	61.01	11.85	0.08
	Primary	38	54.39	12.02	
	Secondary/HS	13	61.22	7.68	
	Graduation	35	55.71	9.21	
Social Relationships Domain	Illiterate	14	58.33	6.54	0.196
	Primary	38	53.51	10.00	
	Secondary/HS	13	52.56	6.26	
	Graduation	35	56.43	9.50	
Environment Domain	Illiterate	14	63.39	11.59	0.257
	Primary	38	58.06	11.64	
	Secondary/HS	13	63.70	9.16	
	Graduation	35	61.43	10.82	

Comparison of WHOQOL-BREF Domain Scores by Marital Status

There were no statistically significant variations in any of the WHOQOL-BREF domain ratings when comparing them according to marital status (p > 0.05). Both married and unmarried participants had comparable scores, with unmarried individuals slightly higher in the psychological domain (57.22 vs. 56.57) and married individuals slightly higher in the environment domain (60.92 vs. 59.58) (Table 7).

**Table 7:** Mean comparison of the WHOQOL-BREF domain with marital status SD: standard deviation, N: number of patients; WHOQOL: World Health Organization Quality of Life

Domains	Marital status	N	Mean	SD	One-way ANOVA
Physical Domain	Married	85	54.37	9.77	0.75
	Unmarried	15	55.24	10.00	
Psychological Domain	Married	85	56.57	10.86	0.83
	Unmarried	15	57.22	10.74	
Social Relationships Domain	Married	85	55.10	9.37	0.97
	Unmarried	15	55.00	7.59	
Environment Domain	Married	85	60.92	11.17	0.67
	Unmarried	15	59.583	11.1720	

Comparison of WHOQOL-BREF Domain Scores Across Occupational Categories

The comparison of WHOQOL-BREF domain scores by occupation revealed no significant differences across all domains (p > 0.05). However, descriptively, housewives reported the highest scores in the physical (56.70), psychological (58.68), and environmental (62.76) domains, while government employees scored the lowest across most domains, particularly in social relationships (48.33) (Table 8).

**Table 8:** Mean comparison of the WHOQOL-BREF domain with occupation SD: standard deviation, N: number of patients; WHOQOL: World Health Organization Quality of Life

Domains	Occupation	N	Mean	SD	One-way ANOVA
Physical Domain	Government Job	5	52.14	5.42	0.6
	Private Job	53	54.18	9.65	
	Daily wage	18	53.17	11.93	
	Housewife	24	56.70	9.02	
Psychological Domain	Government Job	5	51.67	6.97	0.58
	Private Job	53	56.45	9.55	
	Daily wage	18	56.02	13.95	
	Housewife	24	58.68	11.52	
Social Relationships Domain	Government Job	5	48.33	9.13	0.21
	Private Job	53	54.72	7.40	
	Daily wage	18	57.87	9.68	
	Housewife	24	55.21	11.48	
Environment Domain	Government Job	5	55.00	15.72	0.25
	Private Job	53	61.50	9.55	
	Daily wage	18	57.29	12.45	
	Housewife	24	62.76	12.12	

Comparison of WHOQOL-BREF Domain Scores Based on Monthly Household Income

The average WHOQOL-BREF domain ratings for people in various socioeconomic categories are shown in Table 9. The physical domain showed a statistically significant distinction (p = 0.02). In comparison to the ≤1 lakh group (48.90), the one to five lakh group had the highest mean score (58.29), indicating a greater physical QoL. Although there were no major variations in the environment (p = 0.07), social relationships (p = 0.69), or psychological (p = 0.25) categories, those with higher incomes often scored higher. These findings show that income and dialysis patients' physical QoL are positively correlated, with less significant effects on other aspects of QoL (Table 9).

**Table 9:** Mean comparison of the WHOQOL-BREF domain with income SD: standard deviation, N: number of patients; WHOQOL: World Health Organization Quality of Life

Domains	Income	N	Mean	SD	One-way ANOVA
Physical Domain	≤ 1	13	48.90	9.60	0.02
	1 - 5	25	58.29	10.80	
	5 - 10	22	51.79	9.53	
	≥10	16	53.57	6.78	
Psychological Domain	≤ 1	13	51.92	13.13	0.25
	1 - 5	25	58.33	10.55	
	5 - 10	22	57.39	9.53	
	≥ 10	16	53.91	9.06	
Social Relationships Domain	≤ 1	13	57.05	10.68	0.69
	1 - 5	25	54.00	7.65	
	5 - 10	22	55.68	6.50	
	≥ 10	16	54.17	9.62	
Environment Domain	≤ 1	13	53.61	12.09	0.07
	1 - 5	25	60.75	10.01	
	5 - 10	22	63.35	9.21	
	≥ 10	16	59.77	11.57	

Impact of Place of Residence on WHOQOL-BREF Domain Scores

The comparison of WHOQOL-BREF domains with residence showed no statistically significant differences across urban, semi-urban, and rural groups (p > 0.05 for all domains). However, urban individuals had marginally superior results in the environmental area (61.33), while rural inhabitants reported slightly greater scores in the psychological (59.47) and social interaction (56.82) domains. Overall, QoL ratings were not much impacted by where one lived (Table 10).

**Table 10:** Mean comparison of the WHOQOL-BREF domain with residence SD: standard deviation, N: number of patients; WHOQOL: World Health Organization Quality of Life

Domains	Residence	N	Mean	SD	One-way ANOVA
Physical Domain	Urban	24	54.46	10.60	0.97
	Semi Urban	65	54.40	8.30	
	Rural	11	55.19	15.60	
Psychological Domain	Urban	24	58.85	9.06	0.27
	Semi Urban	65	55.38	10.99	
	Rural	11	59.47	12.65	
Social Relationships Domain	Urban	24	55.90	9.98	0.65
	Semi Urban	65	54.49	8.97	
	Rural	11	56.82	8.18	
Environment Domain	Urban	24	61.33	11.94	0.94
	Semi Urban	65	60.43	10.22	
	Rural	11	61.08	15.08	

Association Between Clinical Variables and QoL Domains

Participants receiving dialysis for one to five years and ≥5 years reported higher mean scores in the social connection domain (56.46 and 56.67, respectively) than those receiving dialysis for ≤1 year (50.67), with this difference being statistically significant (p = 0.01), according to a comparison of WHOQOL-BREF domain scores across dialysis duration. Dialysis length did not significantly correlate with the other areas of the environment (p = 0.97), psychology (p = 0.25), or physical (p = 0.61) (Table 11).

**Table 11:** Mean comparison of the WHOQOL-BREF domain with duration of dialysis SD: standard deviation, N: number of patients; WHOQOL: World Health Organization Quality of Life

Domains	Duration of dialysis	N	Mean	SD	One-way ANOVA
Physical Domain	≤ 1	25	54.57	6.96	0.61
	1 - 5	40	55.54	11.32	
	≥ 5	35	53.27	9.66	
Psychological Domain	≤ 1	25	57.33	8.44	0.25
	1 - 5	40	58.33	11.32	
	≥ 5	35	54.29	11.50	
Social Relationships Domain	≤ 1	25	50.67	7.18	0.01
	1 - 5	40	56.46	10.25	
	≥ 5	35	56.67	8.03	
Environment Domain	≤ 1	25	60.75	10.25	0.97
	1 - 5	40	61.02	11.61	
	≥ 5	35	60.36	11.46	

Impact of Income and Duration of Dialysis on Quality of Life

The multiple linear regression analysis indicates that duration of dialysis has a statistically significant positive association with QoL (β = 2.811, p = 0.017), suggesting that longer duration is linked to higher QoL scores. In contrast, income per year does not significantly affect QoL (p = 0.89) (Table 12).

**Table 12:** Multiple linear regression analysis of factors affecting QoL t-value: tests each variable’s effect, Sig.: p-value indicating whether that effect is statistically significant (p < 0.05 = significant); QoL: quality of life

Factors	Unstandardized coefficients		Standardized coefficients	t	Sig.
	B	Std. Error	Beta		
Income (Per year)	0.15	1.14	0.02	0.13	0.89
Duration of dialysis	2.811	1.155	0.239	2.433	0.017

## Discussion

CKD, particularly in its ESKD, significantly impairs individuals’ physical, psychological, and social well-being. HD remains the primary mode of renal replacement therapy in India due to limited organ donation rates and financial constraints [18].

In this regard, evaluating HD patients' QoL is crucial for directing all-encompassing and patient-centered therapy approaches. This research evaluated the psychological, physical, social, and biological QoL of patients undergoing maintenance HD in a tertiary care facility in Mangalore using the WHOQOL-BREF questionnaire. It also looked at related clinical and demographic factors.

The research participants' demographic profile showed that most of them were elderly ≥60 years (52%), male (69%), and residents of semi-urban areas (65%). Most were married (85%), and the education levels varied, with 38% having primary education and 35% being graduates. Around 53% of the people worked in the private sector, and 32.9% said they made between ₹1 and ₹5 lakhs a year. Of the patients, 75% had been on dialysis for more than a year, and there were a lot of other health problems, including diabetes (82.1%) and high blood pressure (86.3%). These findings highlight the many difficulties that the HD population faces and show the complex demographic and clinical features of this group.

Among the WHOQL-BREF domains, the physical domain scored the lowest, indicating significant limitations in mobility, pain, and dependence on medical care. This is consistent with findings from Cho et al. (2022) and Al-Mansouri et al. (2021), who reported similar physical challenges among HD patients [19,20]. In the present study, 88% experienced pain, and 91% reported reliance on ongoing medical treatment, reinforcing the urgent need for integrated symptom management.

The psychological domain showed moderate impairment, with 71% of patients reporting frequent negative feelings. This aligns with Hung et al. (2019), who emphasized that the chronic nature of HD is often associated with anxiety, depressive symptoms, and reduced emotional well-being [21]. Despite this, over half reported some degree of life enjoyment and purpose, suggesting partial preservation of mental resilience.

Dialysis time and the social interactions domain were shown to be significantly correlated (p = 0.01). Patients on dialysis for more than one year had higher social QoL scores, likely due to improved coping mechanisms and social reintegration over time. These findings echo the work of Lalo et al. (2022) and Nissen et al. (2018), who reported that chronic disease patients often develop adaptive strategies that enhance social functioning [22,23]. This challenges the assumption that prolonged dialysis invariably leads to social withdrawal and instead indicates a potential for adaptive social functioning with continued treatment.

The study also found that it was significantly associated with QoL in the physical domain (p = 0.02), with the ₹1-5 lakh group reporting higher scores. While this association does not imply causality, it may suggest that patients with moderate financial stability are better able to access resources that support physical well-being, such as nutritional support, transportation, and consistent treatment adherence. This is consistent with research by Zhu et al. (2024), which found that socioeconomic determinants of health significantly influence dialysis patients' QoL [24].

On the other hand, no QoL domain in the current research showed statistically significant relationships with demographic characteristics such as education, age, employment, gender, marital status, and place of residence. This aligns with Chen et al. (2025), who similarly concluded that static sociodemographic characteristics have a limited influence on QoL outcomes compared to disease-related and treatment-related variables [25].

Importantly, regression analysis revealed a statistically significant association between dialysis duration and overall QoL (β = 2.811, p = 0.017). However, given the cross-sectional nature of the study, this relationship should be interpreted as correlational rather than causal. Long-term prospective studies are needed to confirm whether increased dialysis duration directly improves QoL. This supports the hypothesis that patients may adapt psychologically and functionally over time, stabilizing their routines and experiences. Elias et al. (2025) corroborated this, noting that long-term HD patients often demonstrate better psychological adjustment and structured daily living [26]. Although a few Indian studies have reported impaired QoL among HD patients, direct comparison remains challenging due to heterogeneity in dialysis prescription, study design, and the use of different QoL instruments. Where comparable, our findings concur in showing significant deficits in physical and psychological health domains. Importantly, our study suggests that patients with longer dialysis duration may adapt over time, leading to relatively better QoL in certain domains that have received less emphasis in Indian literature. Clinically, this underscores the need for targeted psychosocial interventions and counselling early in the course of dialysis, when the QoL impact is most pronounced, while reinforcing the importance of sustained support to help patients adapt functionally and psychologically.

Despite these significant findings, our study has several limitations. First, the cross-sectional design precludes longitudinal assessment of changes in QoL and therefore restricts causal inference. Second, as the study was conducted in a single tertiary-care center, the findings may not be generalizable to other geographic or healthcare settings with different resources or dialysis practices. Third, the use of self-reported questionnaires may have introduced recall and response bias, while smaller subgroup sizes (e.g., younger patients or government employees) reduced the statistical power for subgroup analyses. These limitations highlight the need for future multicentric, longitudinal studies with larger and more diverse cohorts. Incorporating qualitative approaches would also allow a deeper understanding of patients’ lived experiences and psychosocial challenges, thereby generating more comprehensive insights into QoL trajectories among individuals receiving maintenance HD.

## Conclusions

This study shows that patients receiving maintenance HD experience an overall mediocre QoL, with the physical and psychological domains being most adversely affected. Beyond the clinical burden, patients reported persistent pain, psychological distress, and treatment-related dependency, reflecting the multidimensional impact of HD. Although longer dialysis duration appeared to facilitate social adaptation, and higher income was associated with better physical health, most demographic variables had limited influence on QoL. These findings highlight the need for integrated care strategies that go beyond routine medical management to incorporate psychosocial support and socioeconomic assistance. Regular QoL assessments in dialysis units may help identify vulnerable patients early and guide tailored interventions aimed at improving long-term outcomes.
